# Complex post-traumatic stress disorder: a much needed diagnosis

**DOI:** 10.1192/bjo.2022.566

**Published:** 2022-09-30

**Authors:** Marylene Cloitre

**Affiliations:** National Center for PTSD, Division of Dissemination and Training, Palo Alto Veterans Association Healthcare System, California, USA; and Department of Psychiatry and Behavioral Sciences, Stanford University, California, USA

**Keywords:** Post-traumatic stress disorder, refugees, human rights, mental health, asylum seekers

## Abstract

This commentary conveys appreciation for a recent review of the rates of complex post-traumatic stress disorder (CPTSD) among refugees, describes the relevance of CPTSD to the refugee experience and discusses implications for assessment and treatment, the effective development of which requires collaboration among researchers, clinicians and individuals with lived experience.

As reported by the United Nations (UN), by the end of 2021 more than 89 million individuals had been forcibly displaced as a result of persecution, conflict, violence or human rights violations, an increase of 6 million from the previous year's total.^1^ This is a record high and, given recent world events, the number of refugees is likely to grow and have an impact on hosting nations. Refugees typically experience multiple types of traumatic stressor across a sustained period of time. Many have experienced repeated violence or human rights violations in the context of ‘minority stress’ related to being discriminated against or stigmatised for their race or ethnicity, a circumstance that has often been ongoing for years, decades or even generations. The opportunity or necessity for migration creates exposure to threat to life and almost always uncertainty about reaching safety. The post-migration period brings its own unique struggles, including fear for the safety of family members, traumatic grief and guilt for family members who have not survived, loss of culture and community, a dislocated sense of self, difficulties finding employment, and risk of stigmatisation and exploitation in the new environment. These experiences are recognised risk factors for mental health problems and in particular for complex post-traumatic stress disorder (CPTSD).^2^ The review by de Silva et al^3^ is significant because it provides an updated account of the likely prevalence of CPTSD among refugees based on the highest quality and most recent studies available, yielding insights into important next steps in estimating and understanding rates of CPTSD in refugees and implications for mental health services.

## Inclusion in ICD-11

In the new ICD-11, released in 2018, the World Health Organization (WHO) included, for the very first time, the diagnosis of CPTSD alongside the more well-established diagnosis of PTSD in the ICD.^4^ This was motivated by the observed need to better account for the different characteristics and consequences of exposure to chronic, sustained and multiple forms of trauma^5^ frequently reported by clinicians. For example, a WHO survey of 3222 mental health providers from 35 countries found that CPTSD was the most frequent diagnosis suggested for inclusion in ICD-11.^6^ The introduction of an empirically supported CPTSD diagnosis in the ICD and the development of a reliable and valid measure^7^ that has been translated into over 30 languages (see www.traumameasuresglobal) has been critically important. The ICD is the diagnostic system that is used worldwide by the UN to identify the prevalence of both physical and mental disorders and to determine the relative distribution of resources (e.g. mental health providers, training programmes) across the globe.

## Prevalence, risk factors and symptom profile

De Silva and colleagues report a CPTSD prevalence rate ranging from 16% to 38% for treatment-seeking samples and from 2.2% to 9.3% in population samples.^3^ Notably, the 15 studies included in the analyses represent three different definitions of CPTSD: ICD-11 CPTSD, the ICD-10 diagnosis of enduring personality change after catastrophic events (EPCACE) and the DSM-IV symptom group referred to as disorder of extreme stress not otherwise specified (DESNOS). Given the recent advances in establishing the validity and reliable measurement of the ICD-11 CPTSD diagnosis, future research is likely to use this diagnosis and the range observed in prevalence rates is more likely to reflect true variation in symptom expression rather than differences in definition or measurement.

A limited but growing number of studies have identified risk factors for CPTSD among refugees. Interpersonal traumas and childhood adversities that pre-date the reason for forced migration contribute to risk for CPTSD^8^ and post-migration problems in living, such as uncertainty about visa status^9^ and low social support,^10^ have been associated with severity of CPTSD symptoms. Moreover, specific environmental factors have been found to contribute to specific symptom groups. For example, experiences of discrimination have been found to have a strong association with negative self-concept, while residency instability has been associated with re-experiencing symptoms.^11^ These results highlight the complex dynamics between history of trauma, the presence of ongoing stressors and trauma-related mental health symptoms.

Network analyses of epidemiological and clinical samples across several countries and languages have revealed a relative invariance in the strength of the relationship of CPTSD symptoms to one another, speaking to the coherence of the CPTSD profile, and have identified the centrality of certain symptoms, with those related to identity often emerging as most salient.^12^ It remains to be seen whether a similar stability in symptoms will be observed across diverse refugee populations and whether the disturbances in identity or self-concept remain a central and organising feature in the CPTSD symptom constellation. This would not be surprising given that refugees are the targets of violence specifically due to their identity, including their race, ethnicity, beliefs and culture, and all of these continue to be disrupted through the post-migration period.

## Assessment and treatment

It is hoped that the ICD-11 CPTSD diagnosis provides a phenomenologically accurate and clinically meaningful characterisation of the symptoms and problems of refugees. The above few examples indicate the necessity of conducting assessments that include history of trauma as well as ongoing stressors and current resources to develop optimally therapeutic treatment plans. A full picture of the individual's health status requires not only diagnostic assessment but also an evaluation of the severity of CPTSD symptoms, the presence of dissociation, anxiety, depression, psychosis and substance misuse, and psychosocial functioning.^8,13^ In addition, refugees often suffer from co-occurring chronic and unattended physical health problems that need to be assessed and addressed.

The development of meaningful assessment and effective treatment plans needs to take into account the specific history and context of culturally diverse refugee populations. These factors shape the expression of psychological distress, as well as perceptions of mental health interventions and pathways to healing and recovery.^8^ Collaboration with individuals with ‘lived experience’ or with organisations that represent refugees is critical in the development of assessments and treatments that are sensitive to culturally diverse populations. Mutually respectful partnerships between relief services and refugee populations are necessary for the creation of effective assessment, triage and treatment. The role of post-migration living difficulties as contributors to mental health status has implications for effective treatment planning and the recovery process. Optimally, mental health services will be sensitive to ongoing stressors such as visa status and residency stability, provide resources and opportunities to enhance functioning and self-determination, and be embedded in social networks and communities that reflect the values, traditions and satisfactions of the individual's culture.

## Pointing to the future

As de Silva and colleagues themselves indicate, their review has some limitations, many of which are due to the current state of the field. These include the limited number of studies, inadequate sample sizes, biases in sample selection, bias in response rates and variation in the definition of CPTSD. Nevertheless, the study provides a scholarly review of the current available data and in doing so, clearly highlights the gaps in the literature and important future directions for CPTSD research among refugees.

## Data Availability

Data availability is not applicable to this article as no new data were created or analysed in this study.
